# Sexualisierte Gewalt in der Erfahrung Jugendlicher: Ergebnisse einer repräsentativen Befragung

**DOI:** 10.1007/s00103-021-03430-w

**Published:** 2021-10-04

**Authors:** Christiane Erkens, Sara Scharmanski, Angelika Heßling

**Affiliations:** Abteilung S – Sexualaufklärung, Verhütung und Familienplanung, Referat S3 – Aufgabenkoordinierung, Nationale und internationale Zusammenarbeit, Forschung und Fortbildung, Bundeszentrale für gesundheitliche Aufklärung (BZgA), Maarweg 149–161, 50825 Köln, Deutschland

**Keywords:** Prävalenz, Junge Menschen, Sexueller Missbrauch, Peers, Disclosure, Prevalence, Young people, Sexual abuse, Peers, Disclosure

## Abstract

**Hintergrund:**

Seit 1998 erhebt die Bundeszentrale für gesundheitliche Aufklärung (BZgA) im Rahmen ihrer Repräsentativbefragung „Jugendsexualität“ Daten zur Verbreitung von sexualisierter Gewalt im Jugendalter. Seither wurde das Messinstrument stetig weiterentwickelt und kann somit auch einen Beitrag zur Bearbeitung der Forschungslücken im Bereich der Prävalenzforschung leisten.

**Ziel:**

Anhand der Ergebnisse der 9. Trendwelle sollen die Prävalenzen erlebter körperlicher und nichtkörperlicher sexualisierter Gewalt in der Erfahrung junger Menschen sowie Daten zu Täterkreisen und zum Disclosure-Verhalten Betroffener dargestellt werden.

**Methode:**

An der kombiniert mündlich-schriftlichen CAPI-Befragung (Computer-assisted Personal Interviewing) nahmen Jugendliche (14–17 Jahre) und junge Erwachsene (18–25 Jahre) teil (*N* = 6032). Der Fragenkatalog wurde im Rahmen der 2019 durchgeführten 9. Welle u. a. um die Frage nach Lebenszeitprävalenzen zu nichtkörperlicher Gewalt erweitert. Erste Ergebnisse werden hier deskriptiv dargestellt.

**Ergebnisse:**

Sexualisierte Gewalt im Jugendalter wird mehrheitlich innerhalb der eigenen Peergruppe (unter gleichaltrigen Bekannten) erfahren. Auch hinsichtlich des Disclosure-Verhaltens betroffener Jugendlichen und jungen Erwachsenen spielen Gleichaltrige eine übergeordnete Rolle.

**Diskussion:**

Die Daten bestärken Ergebnisse anderer Dunkelfeldstudien zu den Unterschieden des Erlebens sexualisierter Gewalt in Kindheit und Jugendalter. Die vorliegende Studie trägt zu einem kontinuierlichen Monitoring bei und kann auch zukünftig sexualisierte Gewalt in der aktuellen Generation junger Menschen erfassen. Es gilt, den Studienergebnissen gezielte, evidenzbasierte und zielgruppenspezifische Präventionsmaßnahmen anzuschließen.

## Abstract (en)

**Background:**

The Federal Centre for Health Education (BZgA) has generated data on the prevalence of sexualised violence in adolescence through its representative survey on youth sexuality since 1998. Since then, the survey has been refined and thus contributes to bridging existing gaps in prevalence research.

**Aim:**

Based on the ninth survey iteration of the youth sexuality study prevalence rates of direct (hands-on) and indirect (hands-off) sexualized violence in adolescence, data on offenders and disclosure of young people after victimization are depicted.

**Methods:**

Computer-assisted personal interviewing (CAPI) was conducted in a sample (*N* = 6032) of adolescents (14–17 years) and young adults (18–25 years). Items on the lifetime prevalence of indirect (hands-off) sexualised violence were included in the questionnaire for the recent wave of the representative survey. This article offers the first descriptive survey results.

**Results:**

Adolescents and young adults experience sexualised violence predominantly within their own peer group. Additionally, peers play a significant role in responding to disclosure of sexualised violence.

**Discussion:**

The findings support results of other population surveys regarding differences between experiences of sexualised violence in childhood and adolescence. The survey is aimed to contribute to continuous monitoring, providing regular datasets on sexualised violence in adolescence. The study aims to induce evidence-based, target-group-specific prevention measures.

## Hintergrund

Die Datenlage zur Prävalenz sexualisierter Gewalt in der Erfahrung Jugendlicher hat sich in der vergangenen Dekade stetig und wesentlich verbessert [1]. Ausgelöst durch die Missbrauchsskandale in pädagogischen und kirchlichen Kontexten und ihrer Aufarbeitung hat 2010 ein regelrechtes Diskursereignis in der Forschungslandschaft stattgefunden [2]. Seither wurde im Rahmen zahlreicher qualitativer sowie quantitativer Forschungsvorhaben eine breite Datenbasis im Themenfeld sexualisierte Gewalt erarbeitet [1, 3–6].^1^

Trotz dieses enormen Wissenszugewinns lassen sich über die Entwicklungen der Verbreitung von sexualisierter Gewalt im Jugendalter kaum Aussagen treffen, da es in Deutschland nach wie vor an einer regelmäßig wiederholten Dunkelfeldstudie mangelt, die sich explizit und ausschließlich mit den Erfahrungen Jugendlicher mit sexualisierter Gewalt beschäftigt [1]. Vor diesem Hintergrund hat die Bundeszentrale für gesundheitliche Aufklärung (BZgA) den Fragenkatalog zu sexualisierter Gewalt in ihrer seit 1980 regelmäßig durchgeführten Befragung zur Jugendsexualität in der aktuellsten Befragungswelle deutlich ausgeweitet.

Im Rahmen der Jugendsexualitätsstudie werden seit 1980 Jugendliche im Alter von 14–17 Jahren – und seit 2015 auch junge Erwachsene zwischen 18 und 25 Jahren – zu Themen der Sexualaufklärung, zu sexuellen Erfahrungen und Verhütung befragt. 1998 wurden erstmals auch Kernfragen zu Erfahrungen Jugendlicher mit sexualisierter Gewalt in ihrer engen Hands-on-Definition im Sinne einer Lebenszeitprävalenz in den Fragenkatalog integriert [7]. Im Fokus steht dabei die Perspektive der Betroffenen. Analog zu den Entwicklungen in Forschung und Praxis hinsichtlich einer Schärfung von Begrifflichkeiten und einer differenzierteren Darstellung der Lebenswelten Jugendlicher wurden auch die Fragestellungen in der vorliegenden Wiederholungsbefragung im Laufe der Jahre maßgeblich angepasst [8].

In diesem Beitrag werden Ergebnisse der 9. Trendwelle der Studie „Jugendsexualität“ aus dem Jahr 2019 vorgestellt. Die Prävalenzen erlebter körperlicher und nichtkörperlicher sexualisierter Gewalt in der Erfahrung junger Menschen sowie Daten zu Täterkreisen und zum Offenbarungsverhalten (Disclosure-Verhalten) Betroffener werden dargestellt.

## Methoden

Die Befragung zu Erfahrungen sexualisierter Gewalt ist eingebettet in eine repräsentative Wiederholungsbefragung zum Sexual- und Verhütungsverhalten junger Menschen. In der Trendwelle 2019 erfolgte eine erhebliche Ausweitung dieses Themenkomplexes: Zum einen wurde körperliche sexualisierte Gewalt tiefgehender und detailreicher als in den vorangegangenen Trendwellen exploriert; so sind beispielsweise Fragen zur Art der körperlichen sexualisierten Gewalt, dem Täterkreis und zum Disclosure-Verhalten^2^ nach dem Übergriff im Instrument enthalten. Als Referenz der Bewertung sollten die Befragten per Instruktion die erste Erfahrung mit sexualisierter Gewalt erinnern.^3^

Zum anderen ist das Spektrum der untersuchten sexualisierten Gewalt um den Bereich nichtkörperlicher Gewalterfahrungen erweitert worden. Hierunter werden in der Befragung Hands-off-Taten verstanden, die verbale und schriftliche Beleidigungen mit sexuellem Bezug, die Verbreitung von Gerüchten sexuellen Inhaltes sowie Konfrontationen mit sexuellen Handlungen und Viktimisierung (Zum-Opfer-Machen) im Internet umfassen. Hier wurde auf ein etabliertes Instrument zur Erfassung zurückgegriffen [9]. Ein weiterer Befragungsfokus lag auf der Bekanntheit einschlägiger Hilfsangebote.

Die Datenerhebung wurde im Zeitraum von Mai bis Oktober 2019 vom Feldinstitut Kantar GmbH mit der CAPI-Methode (Computer-assisted Personal Interviewing) als kombiniert mündlich-schriftliche Interviews durchgeführt. Die Befragung fand in der häuslichen Umgebung der Jugendlichen bzw. jungen Erwachsenen und meistens ohne Anwesenheit Dritter statt. Die Fragen zu erlebter sexualisierter Gewalt beantworteten die Befragten am Laptop (Selbstausfüllerteil) unter Anwesenheit der Interviewerinnen und Interviewer.

Sowohl die Erziehungsberechtigten als auch die Jugendlichen bzw. jungen Erwachsenen wurden im Vorfeld umfassend über Ziel und Zweck der Studie sowie die Datenverarbeitung schriftlich und mündlich aufklärt; die Befragung erfolgte nur nach Einwilligung der Eltern und Jugendlichen bzw. jungen Erwachsenen.

Eine intensive Schulung durch Fachkräfte vor Durchführung der Befragung stellte sicher, dass die Interviewerinnen und Interviewer die Befragung altersangemessen, kultursensibel und empathisch durchführen konnten. Auf weiterführende Beratungsangebote – wie die (anonyme) Beratung in Beratungsstellen oder Telefonhotlines und Websites – konnten die Interviewerinnen und Interviewer verweisen. Im Anschluss an die Befragung erhielten alle Jugendlichen Informationen über sexualisierte Gewalt und einschlägige Hilfsangebote.

Da die Stichproben der Jugendsexualitätsstudie an anderer Stelle bereits beschrieben wurden [10], wird hier auf eine erneute Beschreibung verzichtet.

Im vorliegenden Bericht werden erste deskriptive Ergebnisse^4^ zur Verbreitung von körperlicher und nichtkörperlicher sexualisierter Gewalt für eine repräsentative Stichprobe aus Jugendlichen und jungen Erwachsenen berichtet. Es schließen sich detailliertere Analysen zur Art der erlebten sexualisierten Gewalt, dem Täterkreis sowie dem Disclosure-Verhalten nach dem Übergriff an, wobei zweiseitige *χ*^*2*^-Tests die Häufigkeitsverteilungen auf Signifikanz prüfen. Die statistischen Analysen wurden mit IBM SPSS 25 durchgeführt.

## Ergebnisse

### Häufigkeit von nichtkörperlicher und körperlicher sexualisierter Gewalt

**Figure 1:**
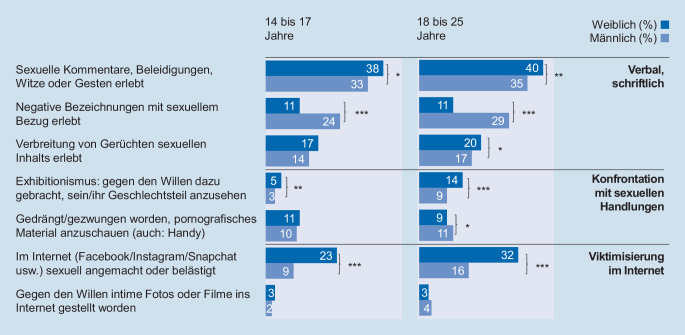

Insgesamt berichten 54 % der befragten Jugendlichen und jungen Erwachsenen, mindestens einmal eine Form von *nichtkörperlicher sexualisierter Gewalt* erlebt zu haben. Männliche und weibliche Befragte sind insgesamt in vergleichbarer Weise betroffen (53 % ggü. 56 %), wobei sich jedoch die Art der Übergriffe zwischen den Geschlechtern unterscheidet (Abb. 1). Mädchen und junge Frauen sind häufiger mit sexuellen Kommentaren (38 % der Jugendlichen/40 % der jungen Erwachsenen), Belästigung im Internet (23 % der Jugendlichen/32 % der jungen Erwachsenen) und Exhibitionismus (5 % der Jugendlichen/14 % der jungen Erwachsenen) konfrontiert, Jungen und junge Männer erleben hingegen mehr negative Bezeichnungen mit sexuellem Bezug (24 % der Jugendlichen /29 % der jungen Erwachsenen).

*Körperliche sexualisierte Gewalt* hat knapp jede fünfte Jugendliche oder junge Frau bereits einmal in ihrem Leben erlebt:^5^ 18 % der Mädchen und Frauen im Alter zwischen 14 und 25 Jahren berichten dies. Ein weiteres Drittel derer, die Gewalt erlebt haben, war nach eigener Aussage bereits mehrfach körperlicher Gewalt ausgesetzt (6 %). Jungen und junge Männer geben mit 5 % eine im Vergleich geringere Betroffenheit an (mehrmalige Betroffenheit: 1 %).

Nachfolgend werden detaillierte Merkmale der ersten erlebten körperlichen sexualisierten Gewalterfahrung dargestellt. Aufgrund dessen, dass männliche Befragte eine geringere Betroffenheit angeben und somit die Fallzahlen für differenzielle Analysen i. d. R. zu klein sind, liegt der Analysefokus überwiegend auf der Stichprobe der Mädchen und jungen Frauen zwischen 14 und 25 Jahren.

### Merkmale der ersten erlebten körperlichen sexualisierten Gewalt

#### Alter bei der ersten erlebten körperlichen sexualisierten Gewalt

Nach Aussage der Mädchen und jungen Frauen fanden mehr als 66 % der ersten erlebten Übergriffe in minderjährigem Alter statt; weitere 16 % der Befragten berichten, jünger als 14 Jahren alt gewesen zu sein (Tab. 1).

**Table 1:** 

	Häufigkeit	Anteil in Prozent
Jünger als 14 Jahre	83	15,7
14 bis 17 Jahre	265	50,5
18 Jahre und älter	158	30,1
Nicht erinnert, keine Angabe	19	3,6
Gesamt	525	100,0

Quelle: eigene Darstellung

#### Art und Täterkreis der ersten erlebten körperlichen sexualisierten Gewalt

In der Stichprobe der 14- bis 25-jährigen Mädchen und jungen Frauen geben 34 % an, dass sie sexualisierte Gewalt in Form von ungewollten körperlichen Berührungen (wie Küssen, Petting) erlebt haben. Weitere 23 % berichten von erzwungenem Geschlechtsverkehr und 17 % von anderen ungewollten sexuellen Handlungen. 37 % der Befragten konnten den Übergriff nach eigenen Angaben abwehren und es kam zu keinen erzwungenen sexuellen Handlungen.^6^

**Figure 2:**
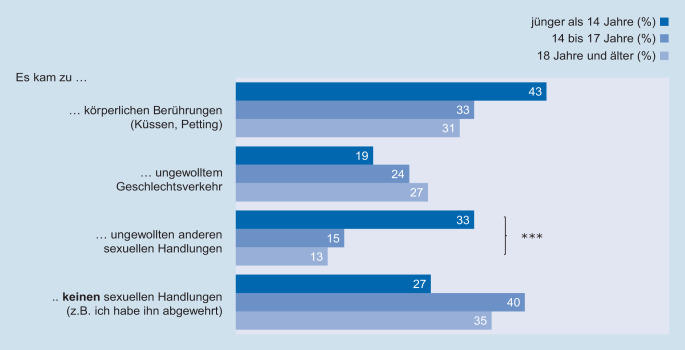

Wie in Abb. 2 dargestellt unterscheidet sich die Art der ersten erlebten sexualisierten Gewalt nicht signifikant in Abhängigkeit von dem Alter, in dem sich der Übergriff ereignete. Lediglich bei Gewalterfahrungen im Kindesalter berichten die Befragten signifikant häufiger von ungewollten anderen sexuellen Handlungen. Tendenziell kommt es im Kindesalter auch häufiger zu ungewollten körperlichen Berührungen wobei dieser Befund nicht signifikant ist.

In Bezug zum Täterkreis zeigen die Daten der Jugendsexualitätsstudie, dass Mädchen und junge Frauen fast ausschließlich sexualisierte körperliche Gewalt durch männliche Täter erleben (97 %). Anders die Aussagen der betroffenen Jungen bzw. jungen Männer: Von ihnen hat die eine Hälfte sexualisierte Gewalt durch Mädchen oder Frauen erlebt (51 %), die andere Hälfte durch Jungen bzw. Männer (49 %).

**Figure 3:**
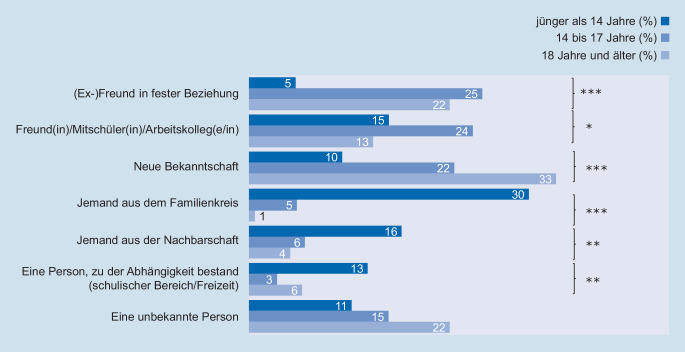

Soll die Beziehung zwischen betroffener Person und Täter bzw. Täterin näher betrachtet werden, so wird hier wieder ausschließlich die Stichprobe der weiblichen Befragten zur Analyse herangezogen. Wie Abb. 3 zu entnehmen ist, unterscheidet sich die Beziehung in Abhängigkeit vom Alter, in dem die Befragten die erste sexualisierte Gewalt erlebten. Bei Übergriffen, die sich im jugendlichen und volljährigen Alter ereigneten, ist der Täter bzw. die Täterin signifikant häufiger der (Ex‑)Freund bzw. die (Ex‑)Freundin, ein Freund/Mitschüler bzw. eine Freundin/Mitschülerin, aber auch eine neue Bekanntschaft. Bei sexualisierter Gewalt im Kindesalter hingegen stammen die Täter und Täterinnen häufiger aus dem sozialen Nahraum, wie beispielsweise eine Person aus dem Familienkreis, der Nachbarschaft oder einem Abhängigkeitsverhältnis im schulischen oder Freizeitbereich.

**Figure 4:**
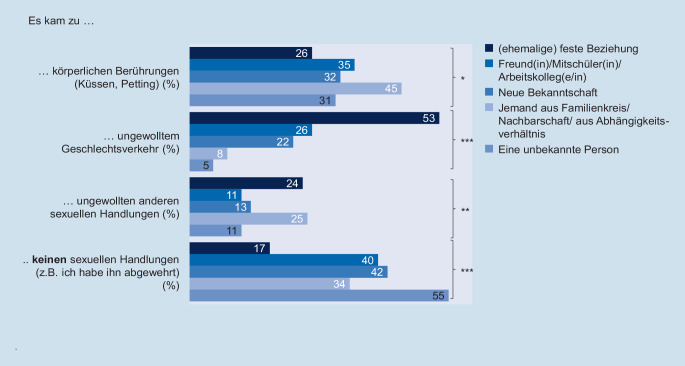

Auffällig ist des Weiteren, dass sich die Art der erlebten sexualisierten Gewalt in Abhängigkeit von der Beziehung zum Täter bzw. zur Täterin unterscheidet (Abb. 4). Geben die Befragten an, dass sich die sexualisierte Gewalt in einer (ehemaligen) Beziehung ereignet hat, so kam es signifikant häufiger zu erzwungenem Geschlechtsverkehr als bei anderen Täterkreisen. War der Täter bzw. die Täterin den Betroffenen hingegen unbekannt, so berichten die Betroffenen signifikant häufiger, dass sie keine erzwungenen sexuellen Handlungen erlebt haben, z. B. weil sie den Täter abwehren konnten.

#### Disclosure nach der ersten erlebten körperlichen sexualisierten Gewalt

Auch die Offenbarung nach der ersten erlebten körperlichen sexualisierten Gewalt wurde in der Jugendsexualitätsstudie erfragt.^7^ Hier zeigt sich, dass ein Viertel der Befragten bisher noch niemandem von dem Übergriff erzählt hat (25 %) und weitere 17 % haben erst nach Jahren über das Erlebte gesprochen. 12 % der Betroffenen haben sich nach einigen Wochen bzw. einigen Monaten offenbart. Knapp die Hälfte hat einige Tage später (17 %) oder unmittelbar danach (29 %) über den erlebten Übergriff gesprochen.

**Figure 5:**
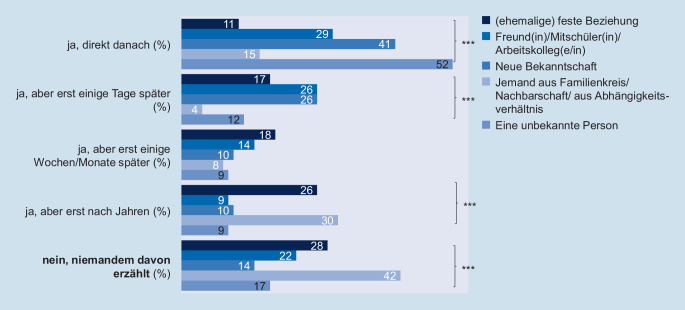

Wie die Daten der Jugendsexualitätsstudie zeigen, ist auch das Disclosure-Verhalten nach der ersten erlebten körperlichen sexualisierten Gewalt von der Beziehung zum Täter bzw. zur Täterin abhängig (Abb. 5). Kommt der Täter bzw. die Täterin aus dem sozialen Nahraum und besteht eine Abhängigkeit und/oder soziale Beziehung zu dieser Person, so geben die Befragten signifikant häufiger an, dass sie noch niemandem von dem Übergriff erzählt haben. Ist der Täter bzw. die Täterin hingegen eine unbekannte Person, so sprechen die Betroffenen vermehrt unmittelbar nach dem Übergriff über das Erlebte.

**Figure 6:**
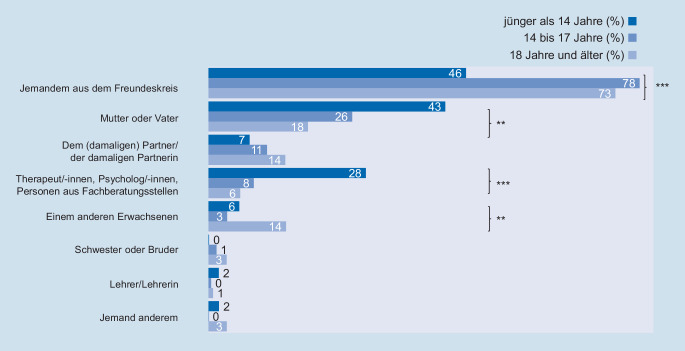

Und auch die Personen, denen sich die Betroffenen nach der ersten erlebten sexuellen Gewalt offenbaren, ist vom Alter zum Zeitpunkt des Übergriffs abhängig. Bei Gewalterfahrungen im Alter von 14 bis 25 Jahren ist die Präferenz eindeutig: Die deutliche Mehrheit wendet sich vor allem an Gleichaltrige (Abb. 6). Eltern, Lehrkräfte und andere Erwachsene spielen bei Offenbarungen von sexualisierter Gewalt im Jugendlichen- und jungen Erwachsenenalter eine untergeordnete Rolle. Aber auch bei Übergriffen im Kindesalter wenden sich die Betroffenen am häufigsten an Gleichaltrige, wenn auch seltener als in den anderen Alterskohorten. Neben Gleichaltrigen offenbaren sich betroffene Kinder dann signifikant häufiger gegenüber Erwachsenen, wie den Eltern oder auch Therapeutinnen und Therapeuten sowie Fachkräften.

## Diskussion

Die 9. Welle der Jugendsexualitätsstudie liefert aktuelle Daten zu sexualisierter Gewalt in der Erfahrung Jugendlicher und junger Erwachsener. Erste Ergebnisse der Studie zur Verbreitung von körperlicher und nichtkörperlicher sexualisierter Gewalt sind hier auf deskriptiver Ebene dargestellt. Vertiefende, multivariate Analysen sind indiziert, um Art und Stärke möglicher Zusammenhänge zwischen mehreren Merkmalen untersuchen zu können.

### Limitationen

Einschränkend sei erwähnt, dass trotz der großen Gesamtstichprobe nur geringe Fallzahlen an Befragten angaben, zum Zeitpunkt der ersten sexualisierten Gewalterfahrung jünger als 14 Jahre gewesen zu sein. Erfahrungen sexualisierter Gewalt im Kindesalter, unterscheiden sich hinsichtlich des Disclosure-Verhaltens der Betroffenen und der Täterkreise maßgeblich von solchen im Jugendalter. Während Kinder überwiegend von sexualisierter Gewalt im familiären Umfeld und dem sozialen Nahraum betroffen sind, machen Jugendliche und junge Erwachsene vermehrt sexualisierte Gewalterfahrungen durch Gleichaltrige. Jugendliche wenden sich zudem signifikant häufiger an Gleichaltrige, um sich nach Erfahrungen sexualisierter Gewalt zu offenbaren. Da diese Unterschiede relevante Implikationen für Prävention und Intervention mit sich bringen, werden die Angaben zu erlebter sexualisierter Gewalt im Alter unter 14 Jahren dennoch dargestellt.

Mögliche Verzerrungen (Social Desirability Bias, Recall Bias oder Self-Report Bias) durch die Anwesenheit der Eltern der befragten Jugendlichen und jungen Erwachsenen im Haushalt können nicht vollständig ausgeschlossen werden. Dabei ist zu beachten, dass ein Bias im Antwortverhalten vorrangig bei Kindern zu erwarten wäre, bei denen sexualisierte Gewalt vermehrt im familiären Setting erlebt wird. Zudem ist anzunehmen, dass alternative Befragungssettings, wie Schule- oder Freizeitkontext, angesichts der erheblichen Rolle von Peers^8^ für Erfahrungen mit sexualisierter Gewalt im Jugendalter ebenso Risiken für Bias mit sich bringen. Ergänzend müsste bei Befragungen Jugendlicher im schulischen Setting von Clustereffekten sowie relevanten Effekten von Nichtteilnahmen („school-based non-response“) ausgegangen und diese methodisch kontrolliert werden [12]. Für eine – trotz möglicher Bias bestehende – gute Vergleichbarkeit der in verschiedenen Befragungssettings erhobenen Daten spricht die große Nähe der Ergebnisse zu Erfahrungen nichtkörperlicher Gewalt der vorliegenden Studie mit der Studie „Speak!“ [5].

### Implikationen

Trotz oben genannter Limitationen liefern die vorliegenden Daten wertvolle Erkenntnisse für die Weiterentwicklung methodischer Ansätze zur Erhebung von Prävalenzen im Dunkelfeld einerseits und die Konzeption präventiver Maßnahmen in der Lebenswelt der Jugendlichen und ihrer Peers.

#### Kontinuierliches Monitoring von sexualisierter Gewalt im Jugendalter

So ermöglicht es die repräsentative Wiederholungsbefragung der Jugendsexualitätsstudie im Sinne eines kontinuierlichen Monitorings, die Verbreitung von körperlicher und nichtkörperlicher sexualisierter Gewalt in der jeweils aktuellen Generation Jugendlicher und junger Erwachsener zu erfassen und die befragten Altersgruppen (14–17 und 18–25) miteinander zu vergleichen. Zudem ist die Vergleichbarkeit mit anderen regionalen, deutschlandweiten und internationalen Studien [6, 9, 13] gewährleistet. Eine Weiterentwicklung der Messinstrumente in Umfang und Inhalt könnte ein langlaufendes Monitoring im Themenfeld unterstützen. So zeigt die Studie unter anderem, dass der Blick auf die Rolle Jugendlicher als Ausführende sexualisierter Gewalt (Aggressorinnen bzw. Aggressoren), die aus den hohen Anteilen an durch Peers erfahrene Grenzverletzungen und Übergriffe abgeleitet werden kann, stärker in den Blick genommen werden sollte. Zudem könnten zusätzliche Fragen zu mehrfacher Viktimisierung durch verschiedene Täter bzw. Täterinnen wertvolle Informationen zum Zusammenhang von Gewalterfahrungen in Kindheit und Jugend liefern.

#### Evidenzbasierte, zielgruppenspezifische Präventionskonzepte und -maßnahmen

Auf Grundlage der vorliegenden Daten können stetig Veränderungen und zielgruppenspezifische Gefährdungskonstellationen festgestellt werden, die dann in Präventions- und Schutzkonzepten gezielt berücksichtigt werden sollten. So sollten Maßnahmen zur Vorbeugung sexualisierter Gewalt gegen Kinder, die überwiegend im Familienkreis und sozialen Nahraum stattfindet, weiter ausgeweitet werden [2]. Die Befragten Jugendlichen zwischen 14 und 17 Jahren hingegen gaben zu 24 % an, sexualisierte Gewalt durch jemanden aus dem eigenen Freundkreis, der eigenen Schule oder durch Arbeitskolleginnen und -kollegen erlebt zu haben. Weitere 25 % dieser Altersgruppe haben sexualisierte Gewalterfahrungen durch ihren (Ex‑)Partner oder ihre (Ex‑)Partnerin erlebt. Jugendlichen widerfährt sexualisierte Gewalt – anders als Kindern – also vermehrt innerhalb der eigenen Peergroup. Dabei sollten auch die Unterschiedlichkeiten hinsichtlich des Geschlechts der Täter bzw. Täterinnen in der Erfahrung weiblicher und männlicher Befragter Beachtung finden.

Während Präventionsmaßnahmen für Kinder auf den Schutz vor Viktimisierung durch Erwachsene und die Befähigung zum Hilfeholen bei erwachsenen Vertrauenspersonen abzielen [1], müssen Maßnahmen zur Prävention sexualisierter Peergewalt die Lebenswelten der Jugendlichen explizit berücksichtigen und stärker partizipatorisch aufklärend, reflektierend und kompetenzstärkend statt bewahrend konzipiert sein.

Die sensiblen Entwicklungsphasen im Übergang zwischen Kindheit und Jugendalter müssen hierbei besondere Berücksichtigung finden und die altersspezifischen Bedarfe an Schutz und Förderung jeweils ausgewogen sein.

Auch hinsichtlich des Disclosure-Verhaltens bestätigt die vorliegende Studie die Ergebnisse anderer Befragungen zu der besonderen Rolle von Gleichaltrigen für betroffene Jugendliche [5, 6]. Es gilt daher zusätzlich zu primärpräventiven Ansätzen in Peergroups, Jugendliche als mögliche Adressatinnen und Adressaten von Disclosure auf Gespräche mit Peers über erlebte sexualisierte Gewalt vorzubereiten und sie zu befähigen, die betroffenen Peers auf dem Weg ins Hilfesystem zu begleiten [15].

Aktuell mangelt es an Präventionskonzepten zu sexualisierter Gewalt im Jugendalter und sogenannten Bystander-Programmen^9^, die ein eingreifendes Verhalten unter Jugendlichen fördern. Hierzu bedarf es einer vertieften wissenschaftlichen Auseinandersetzung mit den alters- und zielgruppenspezifischen Gelingensbedingungen für partizipative Prävention und Kompetenzstärkung von Jugendlichen und jungen Erwachsenen in Abgrenzung zum Kinderschutz. Bisher sind junge Menschen kaum an der Entwicklung von Schutzkonzepten und präventiver sowie aufklärender Maßnahmen beteiligt [17, 18]. Dies gilt es als wesentlichen Beitrag zur Prävention sexualisierter Gewalt und damit zur Förderung der sexuellen und reproduktiven Gesundheit junger Menschen zu begreifen und zu fördern.

## Footnotes

1 Einen Überblick bietet die Buchreihe „Sexuelle Gewalt und Pädagogik“ von Martin Wazlawik und Arne Dekker (Hrsg.).

2 Disclosure (engl. „to disclose“ = enthüllen, offenlegen, verraten) beschreibt „Prozesse des Erinnerns, Einordnens und Offenlegens von sexualisierter Gewalt“ [11, S. 700].

3 Instruktion: „Bitte denken Sie bei den folgenden Fragen an das *erste Mal*, als jemand versucht hat, Sie gegen Ihren Willen zu körperlichen Berührungen oder sexuellen Handlungen zu bringen.“

4 Alle in diesem Artikel veröffentlichten Ergebnisse werden mit einer Designgewichtung berichtet (kombinierte Regional‑, Geschlechter- und Bildungsgewichte sowie für die Gruppe der Befragten mit Migrationshintergrund Gewichte nach Nationalitätsgruppe).

5 Fragestellung: „Hat schon einmal jemand versucht, Sie gegen Ihren Willen zu körperlichen Berührungen oder sexuellen Handlungen zu bringen, indem Sie unter Druck gesetzt wurden?“; Basis: 14- bis 25-Jährige (*N* = 6032).

6 Fragestellung: „Wie ist die Situation ausgegangen? Kam es zu ...?“ (Mehrfachnennungen); Basis: 14- bis 25-jährige Mädchen/junge Frauen mit sexualisierter Gewalterfahrung (*n* = 538, ungewichtet).

7 Fragestellung: „Haben Sie jemandem von diesem Erlebnis erzählt?“; Basis: 14- bis 25-jährige Mädchen/junge Frauen mit sexualisierter Gewalterfahrung (*n* = 538).

8 Peer (engl. = gleichaltrig, gleichrangig, gleichgesinnt) beschreibt altershomogene Gruppen, deren Angehörige soziale und institutionelle Lebenswelten gemein haben [14].

9 Bystander-Programme (engl. = Umstehende) beschreiben Maßnahmen, die auf die Prävention (sexualisierter) Gewalt im Beisein Dritter, durch die Förderung der Handlungskompetenzen dieser Dritten als Eingreifende abzielen [16].

## References

[1] Kindler H, Derr R (2018). Prävention von sexualisierter Gewalt gegen Kinder und Jugendliche: Fortschritte, gegenwärtiger Stand und Perspektiven.

[2] Kappler S, Hornfeck F, Pooch M‑T, Kinder H, Tremel I (2019) Kinder und Jugendliche besser schützen – der Anfang ist gemacht: Schutzkonzepte gegen sexuelle Gewalt in den Bereichen: Bildung und Erziehung, Gesundheit, Freizeit. https://www.dji.de/fileadmin/user_upload/bibs2019/28116_UBSKM_DJI_Abschlussbericht.pdf. Zugegriffen: 19. Mai 2021 (Abschlussbericht des Monitorings zum Stand der Prävention sexualisierter Gewalt an Kindern und Jugendlichen in Deutschland (2015–2018))

[3] Häuser W, Schmutzer G, Brähler E, Glaesmer H (2011). Misshandlungen in Kindheit und Jugend: Ergebnisse einer Umfrage in einer repräsentativen Stichprobe der deutschen Bevölkerung. Dtsch Arztebl Int.

[4] Posch L, Bieneck S, Baier D, Pfeiffer C (2016). Sexual abuse of children and adolescents: prevalence and trends. Representative studies on victimisation: research findings from Germany.

[5] Maschke S, Stecher L (2018). Sexuelle Gewalt: Erfahrungen Jugendlicher heute.

[6] Hofherr S (2017) Wissen von Schülerinnen und Schülern über sexuelle Gewalt in pädagogischen Kontexten. Kurzbericht über zentrale Ergebnisse. https://www.dji.de/fileadmin/user_upload/bibs2017/hofherr_schuelerwissen_sexuelle_gewalt.pdf. Zugegriffen: 19. Mai 2021

[7] Hessling A, Bode H (2006). Jugendsexualität. Wiederholungsbefragung von 14- bis 17-Jährigen und ihren Eltern.

[8] Hessling A, Bode H (2015). Jugendsexualität 2015. Repräsentative Wiederholungsbefragung. Die Perspektive der 14- bis 25 Jährigen.

[9] Maschke S, Stecher L (2017) Sexualisierte Gewalt in der Erfahrung Jugendlicher. Öffentlicher Kurzbericht. https://www.speak-studie.de/pdf/Kurzbericht%20Speak%20berufliche%20Schulen%20HKM%2026.02.2021.pdf. Zugegriffen: 8. Sept. 2020

[10] Scharmanski S, Hessling A (2021). Sexual- und Verhütungsverhalten von Jugendlichen und jungen Erwachsenen in Deutschland. Aktuelle Ergebnisse der Repräsentativbefragung Jugendsexualität. Bundesgesundheitsblatt Gesundheitsforschung Gesundheitsschutz.

[11] Rieske TV, Scambor E, Wittenzellner U, Retkowski A, Treibel A, Tuider E (2018). Aufdeckungsprozesse – Dimensionen und Verläufe. Handbuch sexualisierte Gewalt und pädagogische Kontexte. Theorie, Forschung, Praxis.

[12] Smit F, Zwart WD, Spruit I, Monshouwer K, Ameijden EV (2002). Monitoring substance use in adolescents: school survey or household survey?. Drugs (Abingdon Engl).

[13] Jud A, Kosirnik C, Mitrovic T (2018). Mobilizing agencies for incidence surveys on child maltreatment: successful participation in Switzerland and lessons learned. Child Adolesc Psychiatry Ment Health.

[14] Köhler S-M, Krüger H-H, Pfaff N (2016). Handbuch Peerforschung.

[15] Gulowski R, Krüger C (2020). Jugendliche reden über sexualisierte Gewalterfahrungen vor allem mit ihren Peers – Erste Erkenntnisse aus dem BMBF-Projekt „Peers als Adressaten von Disclosure und Brücken ins Hilfesystem“.

[16] Banyard VL (2011). Who will prevent sexual violence. Creating an ecological model of bystander intervention. Psychol Violence.

[17] Wolff M, Rusack T, Eßer F, Wazlawik M, Voß H-J, Retkowski A, Henningsen A (2019). Die Organisation von Schutz als alltägliche Praxis. Sexualität und Schutzkonzepte aus der Perspektive von Jugendlichen in stationären Einrichtungen. Sexuelle Gewalt in pädagogischen Kontexten. Aktuelle Forschungen und Reflexionen.

[18] Henningsen A, Winter V (2020). SchutzNorm: Partizipative Forschung im Kontext von Jugendschutz als Bildungsprozess.

